# Supplementary figures and images for: The influence of prosocial priming on visual perspective taking and automatic imitation
Source: PLoS One. 2019 Jan 23;14(1):e0198867. doi: 10.1371/journal.pone.0198867 (PMC6343917; doi:10.1371/journal.pone.0198867)

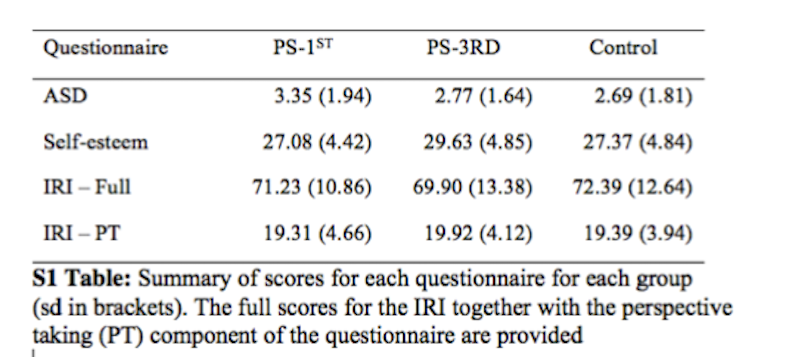

Supplement: S1 Table — (TIFF) [file pone.0198867.s001.tiff]

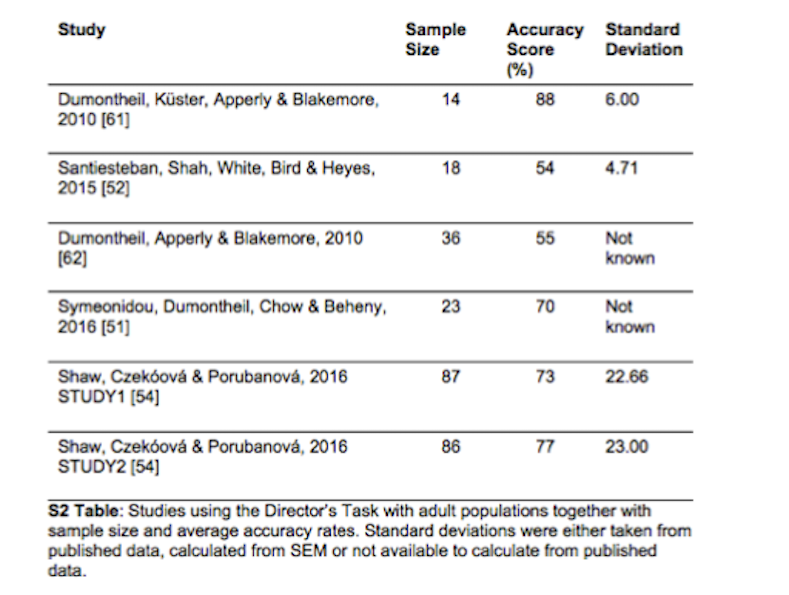

Supplement: S2 Table — (TIFF) [file pone.0198867.s002.tiff]

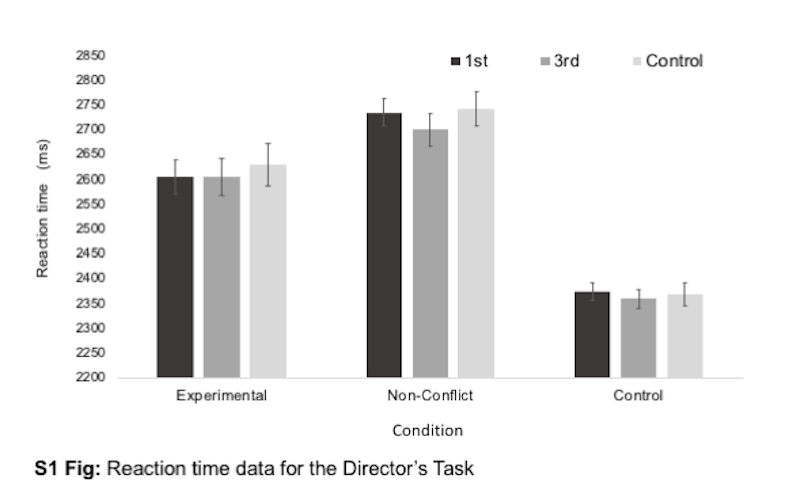

Supplement: S1 Fig — (TIFF) [file pone.0198867.s003.tiff]
